# Promotional effect of silver nanoparticle embedded Ga–Zr-codoped TiO_2_ as an alternative anode for efficient blue, green and red PHOLEDs†

**DOI:** 10.1039/c9ra01025d

**Published:** 2019-05-03

**Authors:** Jayaraman Jayabharathi, Pavadai Nethaji, Venugopal Thanikachalam

**Affiliations:** Department of Chemistry, Annamalai University Annamalainagar 608 002 Tamilnadu India

## Abstract

Efficient blue, green and red phosphorescent OLEDs have been harvested from silver nanoparticles embedded at a glass:Ga–Zr-codoped TiO_2_ interface. The embedded silver nanoparticles at the interface removed the non productive hole current and enhanced the efficiencies. The blue emitting device (456 nm) with emissive layer Ir(fni)_3_ exhibits a maximum luminance (*L*) of 40 512 cd m^−2^ (ITO – 37 623 cd m^−2^), current efficiency (*η*_c_) of 41.3 cd A^−1^ (ITO – 40.5 cd A^−1^) and power efficiency (*η*_p_) of 43.1 lm w^−1^ (ITO – 39.8 lm w^−1^) and external quantum efficiency (*η*_ex_) of 19.4% (ITO – 6.9%). A newly fabricated green device based on emissive layer Ir(tfpdni)_2_(pic) shows intensified emission at 514 nm, luminance of 46 435 cd m^−2^ (ITO – 40 986 cd m^−2^), current efficiency of 49.7 cd A^−1^ (ITO – 47.3 cd A^−1^), power efficiency of 48.6 lm w^−1^ (ITO – 41.4 lm w^−1^) and external quantum efficiency of 17.5% (ITO – 14.9%). The red device (618 nm) with emissive layer Ir(bbt)_2_(acac) shows luminance of 8936 cd m^−2^ (ITO – 8043 cd m^−2^), current efficiency of 6.9 cd A^−1^ (ITO – 4.6 cd A^−1^), power efficiency of 5.7 lm w^−1^ (ITO – 4.9 lm w^−1^) and external quantum efficiency of 9.3% (ITO – 6.9%).

## Abstract 2

Efficient blue, green and red phosphorescent OLEDs have been harvested from silver nanoparticles embedded at a glass:Ga–Zr-codoped TiO_2_ interface.

## Graphical abstract

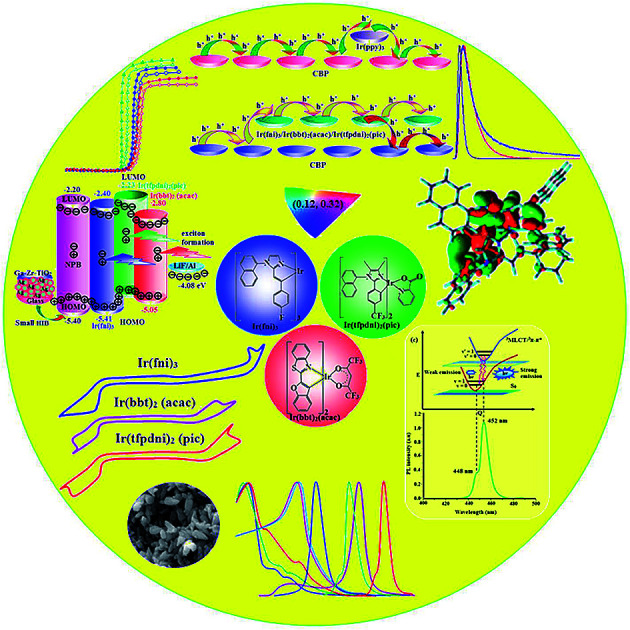

## 1. Introduction

Over the past three decades increasing attention has been paid to applied research in OLEDs due to their potential application in flat-panel displays. The efficiencies of OLEDs can be influenced by the electrodes, constituent materials and interfaces.^1^ The hole injection barrier (HIB) in the device ITO/NPB/Alq_3_/LiF/Al is ≈ 0.6 eV; because of dissociation of metal fluoride, the cathode interface barrier does not exist.^2,3^ In bare ITO anode devices, the energy barrier between ITO and the hole injection layer (NPB) is high resulting in fewer holes being injected. The balanced injected carriers could enhance exciton formation and improving the luminous efficiency (*η*_c_) of the device. Due to the buffer layer at the ITO:NPB interface accumulation of holes at the interface results in effective recombination and improved efficiencies: the LiF coating between the cathode and ETL enhanced the electron injection from the cathode resulting in excellent performances.^4,5^ A wide band gap metal oxide semiconductor used as the hole buffer layer (HBL) (HBL; Si_3_N_4_,^5^ SiO_2_,^6^ Al_2_O_3_,^7^ MoO_3_,^8^ WO_3_,^9^ Pr_2_O_3_,^10^ NiO,^11^ Teflon,^12^ Ta_2_O_5_,^13^ TiO_2_ (ref. 14 and 15) *etc.*) was inserted between ITO and HTL in the interface. The modified ITO surface enhanced the hole injection which promoted the balanced recombination and improved the efficiencies. The operating voltage of OLEDs decreased significantly with increased luminous efficiency (*η*_c_) by using TiO_2_ as the HIB.^15^

Though ITO is chemically unstable, harmful to human and unsuitable with flexible substrates, there is increasing demand of ITO because of its utility in various electronic devices. Therefore, efforts have been made for developing indium-free transparent conducting oxides which should exhibit comparable properties as that of ITO. The electrical resistivity of TiO_2_ thin film can be further improved by doping with B^3+^, Ga^3+^, and Al^3+^: replacing Ti^4+^ ions in a TiO_2_ crystal lattice provide an extra electron^16–18^ which enhanced the optoelectronic properties comparable to that of ITO.^19,20^ Gallium doped TiO_2_ thin film with close Ga–O (1.92 Å) and Ti–O (1.86 Å) bond length in Ga-doped TiO_2_ exhibit high electrochemical stability with minimized crystal. The smooth surface attributed by surfactant effect of Ga^3+^ (74.5 pm) doping in TiO_2_ is beneficial for the development of OLED applications.^18,20^ Compared with group III elements, Zr-doped TiO_2_ exhibits increasing thin film conductivity: Zr and Ti are having same valence state (4+) with similar atom semidiameters (Ti-2.00 Å; Zr-2.16 Å): both semiconductors are n-type with almost identical properties. Therefore, it is possible for substitution of Ti atom by Zr atom in TiO_2_ lattice which introduce lattice defects and Zr-doped TiO_2_ possess high thermo-stabilizing properties.^21^ Therefore, we aimed to synthesize Ga–Zr-codoped TiO_2_ for OLED applications. The surface-plasmon of noble-metal nanostructures enhanced the optoelectronic device efficiencies.^22–37^ The OLED efficiency have been increased by reducing the hole injection barrier through embedding noble-metal nano semiconductors. The interface have been stabilized *via* tuning HIL^38–41^ by forming electrical double layer on anode that stabilized the vacuum level.^38^ To our knowledge is concern, only few reports about efficient red, green and blue OLEDs with TiO_2_ as cost effective anode material instead of ITO. We aimed to combine the advantages of Ga and Zr elemental doping of TiO_2_ as a high performance Ga–Zr-codoped TiO_2_ nanoparticles for OLED applications. We report the efficient red (R), green (G) and blue (B) phosphorescent OLEDs using silver nanoparticles sandwiched between Ga–Zr-codoped TiO_2_ NPs and glass substrate with Ir(bbt)_2_(acac), Ir(tfpdni)_2_(pic) and Ir(fni)_3_ as red, green and blue emitters, respectively.

## 2. Experimental

### 2.1. Characterization

The structure of red, green and blue emitters emissive materials namely Ir(bbt)_2_(acac), Ir(tfpdni)_2_(pic) and Ir(fni)_3_ was confirmed with ^1^H and ^13^C NMR and mass spectra, using Bruker 400 MHz spectrometer and Agilent LCMS VL SD, respectively. Potentials were determined from CHI 630A potentiostat electrochemical analyzer. Optical absorption studies were made bu using Lambda 35 PerkinElmer and Lambda 35 spectrophotometer with integrated sphere (RSA-PE-20) instrument. PerkinElmer LS55 fluorescence spectrometer measurement was employed to study emission characteristics. Thermal characteristics of the materials were analysed with decomposition (*T*_d_) and glass transition (*T*_g_) temperatures recorded with PerkinElmer thermal analysis system (10 °C min^−1^; N_2_ flow rate – 100 ml min^−1^) and NETZSCH (DSC-204) (10 °C min^−1^ under N_2_ atmosphere) instruments, respectively. XPS of nanomaterials were recorded with X-ray photoelectron spectra: ESCA^−3^ Mark II spectrometer-VG – Al Kα (1486.6 eV) radiation. SEM (scanning electron microscopic images) images and EDS (energy dispersive X-ray spectra) of nanomaterials were recorded by using JEOL JSM-5610 equipped with back electron (BE) detector and FEI Quanta FEG, respectively. The TEM (transmission electron microscopy) image of nanomaterials was obtained from Philips TEM with 200 kV electron beam and SAED (selected area electron diffraction) pattern was obtained from Philips TEM (CCD camera; 200 kV). The XRD of nanomaterials was recorded using Equinox 1000 diffractometer (Cu Kα rays; 1.5406 Å; current – 30 mA; 40 kV).

### 2.2. Fabrication of HyLEDs

The newly synthesized iridium(iii) complexes namely, Ir(bbt)_2_(acac), Ir(tfpdni)_2_(pic) and Ir(fni)_3_ are employed as red, green and blue emitters, respectively. The fabricated OLEDs are having the following configuration: glass/Ag (2 nm)/Ga–Zr-codoped TiO_2_ (60 nm)/NPB (40 nm)/CBP:Ir(fni)_3_ (25 nm)/LiF (1 nm)/Al (100 nm) (I); glass/Ag (2 nm)/Ga–Zr-codoped TiO_2_ (60 nm)/NPB (40 nm)/CBP:Ir(bbt)_2_(acac) (25 nm)/LiF (1 nm)/Al (100 nm) (III) and glass/Ag (2 nm)/Ga–Zr-codoped TiO_2_ (60 nm)/NPB (40 nm)/CBP:Ir(tfpdni)_2_(pic) (25 nm)/LiF (1 nm)/Al (100 nm) (IV). The reference devices with the configuration of ITO/NPB (40 nm)/CBP:Ir(fni)_3_ (25 nm)/LiF (1 nm)/Al (100 nm) (II); ITO/NPB (40 nm)/CBP:Ir(bbt)_2_(acac) (25 nm)/LiF (1 nm)/Al (100 nm) (IV) and ITO/NPB (40 nm)/CBP:Ir(tfpdni)_2_(pic) (25 nm)/LiF (1 nm)/Al (100 nm) (VI) have been fabricated. The fabrication was made by using vacuum deposition (5 × 10^−6^ torr) over ITO-coated glass with 20 Ω per square resistance. Organic substances deposition was made on the ITO glass substrates with a rate of 1–2 Å s^−1^ and LiF was evaporated thermally over organic surface. The EL spectra, luminance characteristics and CIE coordinates measured with recorded with USB-650-VIS-NIR spectrometer (Ocean Optics, Inc, USA). Thickness was determined with quartz crystal thickness monitor and current density (*J*)–voltage (*V*) and luminescence (*L*)–voltage (*V*) studies were performed using Keithley 2400 source meter.

### 2.3. Synthesis of Ga–Zr-codoped TiO_2_

To titanium isopropoxide (1.15 g) in 25 ml methoxy ethanol solution, a mixture of gallium nitrate (1.15 g) and zirconium nitrate (1.15 g) was added. The solution was stirred at 150 °C for 1 h. The sol was after cooling which was dried at 180 °C for 24 h and calcinated (at 500 °C: 6 h: heating rate 10 °C min^−1^).^42,43^

### 2.4. Synthesis of silver nanoparticles (Ag NPs)

The *M. elengi* fruit pericarp powder was extracted with water and filtered through 0.22 μm cellulose nitrate membrane filter paper. The extract was stirred with aqueous 10 ml 0.01 M AgNO_3_ at 60 °C for 1 h and Ag NPs solution was stored at 5 °C.^44^

### 2.5. Synthesis of 2-(4-(trifluoromethyl)phenyl)-4,5-dimethyl-1-(naphthalen-1-yl)-1*H*-imidazole (tfpdni)

**Scheme 1:**
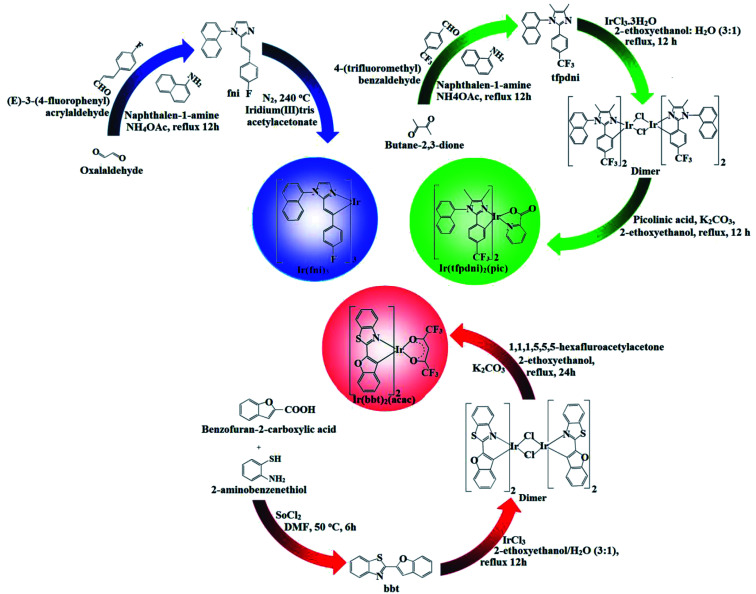
Synthetic route of Ir(fni)_3_, Ir(tfpdni)_2_(pic) and Ir(bbt)_2_(acac).

A mixture of butane-2,3-dione (1 mmol), 4-(trifluoromethyl)benzaldehyde (1 mmol), naphthalen-1-amine (1 mmol) and ammonium acetate (1 mmol) in acetic acid (20 ml) was refluxed (80 °C; 24 h; under N_2_). After cooling, the reaction mixture was extracted with dichloromethane and column chromatographed using benzene : ethyl acetate (9 : 1) as the eluent (Scheme 1). Yield: 63%. Anal. calcd. for C_22_H_17_F_3_N_2_: C, 72.12; H, 4.68; N, 7.65. Found: C, 71.92; H, 4.23; N, 7.15. ^1^H NMR (400 MHz, CDCl_3_): *δ* 2.28 (s, 6H), 7.34 (m, 4H), 7.43–7.41 (d, 2H), 7.52 (d, 2H), 7.78 (m, 3H). ^13^C NMR (100 MHz, CDCl_3_): 4.9, 11.8, 123.82, 124.23, 125.53, 127.37, 127.96, 131.08, 132.49, 134.61, 144.58. MS: *m*/*z*. 366.13 [M^+^] calcd. 364.12.

### 2.6. Synthesis of iridium(iii)-bis(*E*)-2-(4-(trifluoromethyl)phenyl)-4,5-dimethyl-1-(naphthalen-1-yl)-1*H*-imidazole-*N*,C^2^ picolinate [Ir(tfpdni)_2_(pic)]

The tfpdni (2.2 mmol) and iridium(iii) chloride trihydrate (1 mmol) in 2-ethoxyethanol : H_2_O (3 : 1) was refluxed at 120 °C under N_2_ stream. After cooling the precipitated dimer was purified with hexane washings. The dimer (1 mmol) was treated with picolinic acid (2.2 mmol) and potassium carbonate (2.5 mmol) in 2-ethoxyethanol (15 ml) and refluxed at 120 °C under nitrogen stream.^45^ The filtered picolinate iridium(iii) complex Ir(tfpdni)_2_(pic) was purified by washings and the purity of Ir(tfpdni)_2_(pic) complex was checked by HPLC analysis (Fig. S1†) and used as emissive layer without purification by sublimation. Yield: 58%. ^1^H NMR (400 MHz, CDCl_3_): *δ* 2.17 (s, 12H), 7.23 (m, 8H), 7.32 (d, 1H), 7.41 (d, 4H), 7.67 (m, 6H), 7.87 (t, 1H), 8.19 (t, 1H), 8.36 (d, 1H), 9.02 (d, 1H). ^13^C NMR (100 MHz, CDCl_3_): 4.2, 10.9, 123.32, 124.19, 124.70, 125.22, 126.27, 126.96, 127.43, 130.18, 131.19, 133.41, 138.06, 143.58, 148.53, 149.62, 173.12. MALDI-TOF MS: *m*/*z*. 1041.24 [M^+^]; calcd. 1041.08.

### 2.7. Synthesis of 2-(4-fluorostyryl)-1-(naphthalen-1-yl)-1*H*-imidazole (fni)

A mixture of (*E*)-3-(4-fluorophenyl)acrylaldehyde (1 mmol), glyoxal (1 mmol), naphthalene-1-amine (1 mmol) and ammonium acetate (1 mmol) in ethanol (20 ml) was refluxed (12 h; under N_2_ stream). The mixture was cooled, filtered and column chromatographed (benzene : ethyl acetate (9 : 1) as eluent) (Scheme 1). Yield: 65%. Anal. calcd. for C_21_H_15_FN_2_: C, 80.24; H, 4.81; N, 8.91. Found: C, 80.12; H, 4.45; N, 8.87. ^1^H NMR (400 MHz, CDCl_3_): *δ* 6.89 (s, 2H), 6.92 (d, 2H), 7.29–7.41 (m, 7H), 7.60–7.80 (m, 4H). ^13^C NMR (100 MHz, CDCl_3_): 113.82, 116.43, 125.13, 126.37, 128.26, 132.78, 133.49, 135.61, 137.08, 163.06. MS: *m*/*z*. 314.36 [M^+^] calcd. 312.25.

### 2.8. Synthesis of *fac*-tris[2-(4-fluorostyryl)-1-(naphthalen-1-yl)-1*H*-imidazolynato-C^2^,*N*^1^]iridium(iii) [Ir(fni)_3_]

A mixture of 2-(4-fluorostyryl)-1-(naphthalen-1-yl)-1*H*-imidazole (fni) (7.83 mmol), iridium(iii) trisacetylacetonate (1.56 mmol) and glycerol (9 ml) was refluxed (240 °C; N_2_ stream; 48 h). After cooling, the reaction mixture was extracted with dichloromethane, after evaporation the *fac*-isomer Ir(fni)_3_ complex was obtained^46,47^ and purified by hexane washings. The purity of the complex was checked by HPLC analysis (Fig. S1†) and used as emissive layer without purification by sublimation. Yield: 55%. ^1^H NMR (400 MHz, CDCl_3_): *δ* 6.90 (s, 3H), 6.92 (d, 6H), 7.20–7.32 (d, 21H), 7.40 (d, 3H), 7.60 (m, 9H).·^13^C NMR (100 MHz, CDCl_3_): 112.72, 114.43, 124.08, 125.32, 127.16, 130.68, 132.79, 134.41, 136.80, 161.26. MALDI-TOF MS: *m*/*z*. 1236.39 [M^+^]; calcd. 1234.30.

### 2.9. Synthesis of 2-(benzofuran-2-yl)benzo[*d*]thiazole (bbt)

To 2-aminobenzenethiol (1 mmol) in dry DMF, benzofuran-2-carboxylic acid (1 mmol) and SOCl_2_ (10.1 mmol) was added and refluxed at 50 °C for one day. The solvent was removed and the crude solid was washed with a saturated solution of NaHCO_3_, 1 N HCl and cold water. The crude was column chromatographed (hexane : ethyl acetate (7 : 3) as eluent). Yield: 54%. Anal. calcd. for C_15_H_9_NOS: C, 71.69; H, 3.61; N, 5.57. Found: C, 71.32; H, 3.36; N, 5.25. ^1^H NMR (400 MHz, CDCl_3_): *δ* 6.71 (s, 1H), 7.11–7.22 (t, 2H), 7.41–7.56 (m, 4H), 8.14 (d, 1H), 8.25 (d, 1H). ^13^C NMR (100 MHz, CDCl_3_): 102.92, 111.86, 121.27, 121.81, 123.45, 123.90, 124.54, 125.77, 135.35, 153.61, 155.48, 156.10. MS: *m*/*z*. 251.04 [M^+^] calcd. 249.92.

### 2.10. Synthesis of iridium(iii)-bis-2-(benzofuran-2-yl)benzo[*d*]thiazole-*N*,C^2^ acetylacetonate [Ir(bbt)_2_(acac)]

A mixture of 2-(benzofuran-2-yl)benzo[*d*]thiazole (bbt) (2.2 mmol) in 2-ethoxyethanol : H_2_O (3 : 1) was refluxed with iridium(iii) chloride trihydrate (1 mmol) at 120 °C under N_2_ stream. The formed dimer after hexane washings (1 mmol) was refluxed with ancillary ligand 1,1,1,5,5,5-hexafluoropentane-2,4-dione (2.2 mmol) and potassium carbonate (2.5 mmol) in 2-ethoxyethanol (15 ml) at 120 °C under nitrogen stream.^45^ The precipitated iridium(iii) complex was subjected to hexane and petroleum ether washings (Scheme 1). The purity of the complex was checked by HPLC analysis (Fig. S1†) and used as emissive layer without purification by sublimation. Yield: 58%. ^1^H NMR (400 MHz, CDCl_3_): *δ* 1.48 (d, 1H), 1.73 (d, 1H), 4.04 (s, 2H), 5.1 (s, 1H), 7.01–7.11 (t, 3H), 7.30–7.45 (m, 8H), 8.03 (d, 2H), 8.14 (d, 2H). ^13^C NMR (100 MHz, CDCl_3_): 5.2, 65.10, 101.82, 109.96, 111.25, 120.48, 122.54, 122.90, 123.54, 124.67, 134.53, 139.63, 152.16, 154.84, 155.08. MALDI-TOF MS: *m*/*z*. 905.05 [M^+^]; calcd. 904.98.

## 3. Result and discussion

### 3.1. Characterisation of electron injection layer

**Fig. 1:**
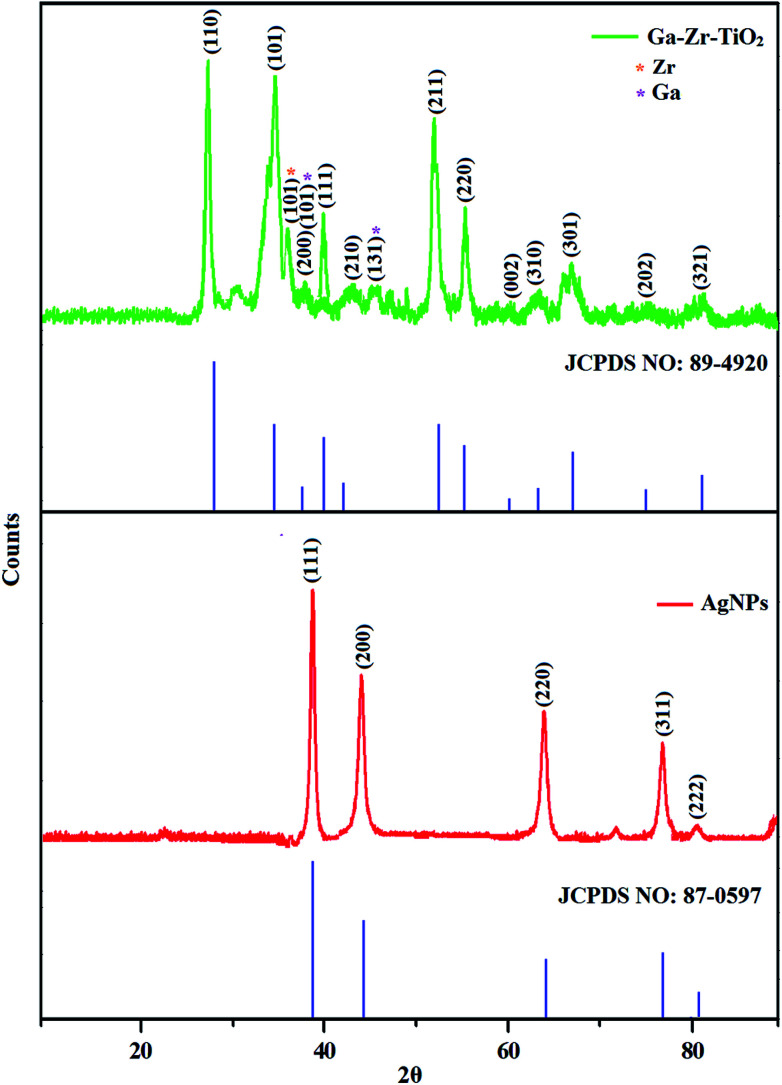
X-ray diffraction pattern of Ga–Zr-codoped TiO_2_ (JCPDS: 89-4920) and Ag NPs (JCPDS: 87-0597).

**Fig. 2:**
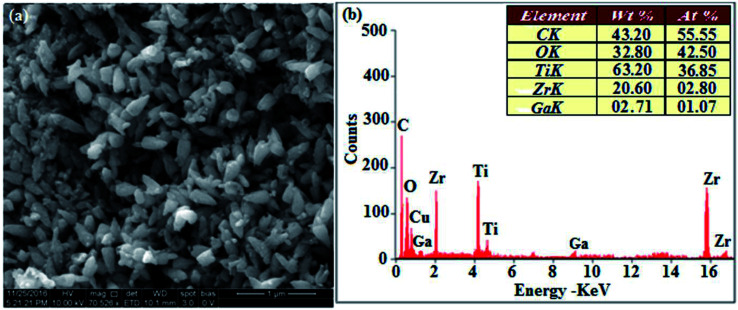
HR-SEM image and EDX of Ga–Zr-TiO_2_.

**Fig. 3:**
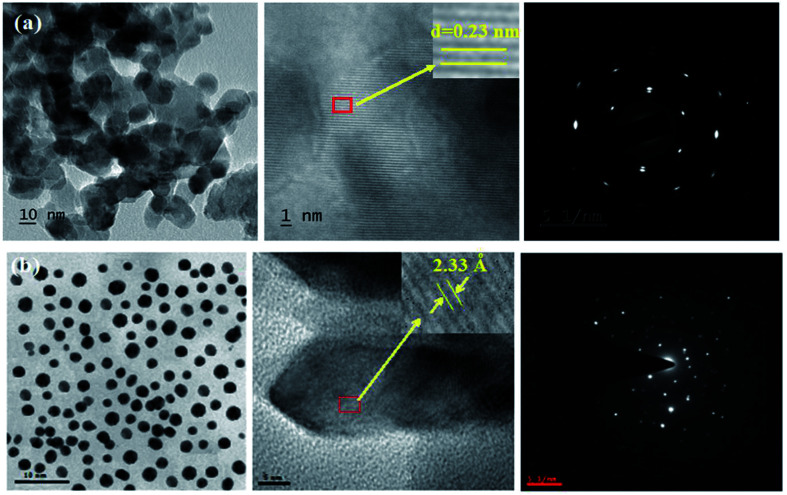
HR-TEM images and SAED pattern of (a) Ga–Zr-codoped TiO_2_ and (b) Ag NPs.

**Fig. 4:**
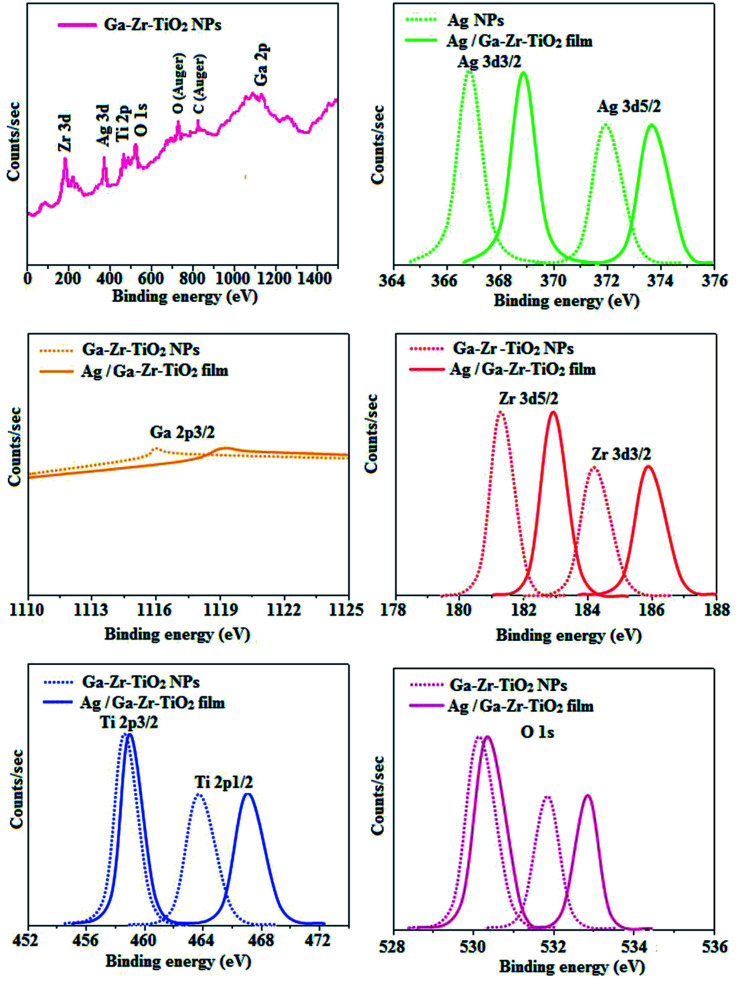
X-ray photoelectron spectra (XPS) of Ga–Zr-codoped TiO_2_ and Ag/Ga–Zr-codoped TiO_2_ film.

**Fig. 5:**
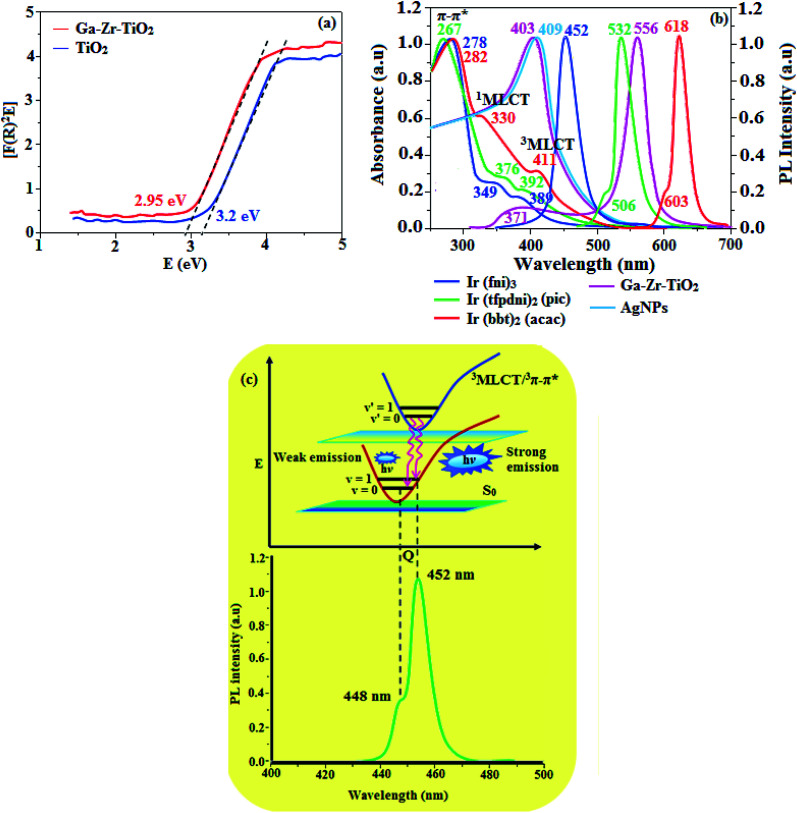
(a) DRS Spectra of TiO_2_ and Ga–Zr-TiO_2_; (b) normalized absorption and emission spectra of Ir(fni)_3_, Ir(tfpdni)_2_(pic) and Ir(bbt)_2_(acac) and (c) Franck–Condon electronic transitions of Ir(fni)_3_.

The XRD pattern of Ga–Zr-codoped TiO_2_ (JCPDS no. 89-4920) and Ag NPs (JCPDS no. 87-0597) is displayed in Fig. 1. The tetragonal crystal structure of Ga–Zr-codoped TiO_2_ with crystal constants *a* and *b* as 4.584 Å and *c* as 2.953 Å exhibit the intensified orientation in (110) plane. The crystal size of Ga–Zr-codoped TiO_2_ was deduced as 11.30 nm and surface area as 93.6 m^2^ g^−1^.^46^ From TEM image of Ga–Ti-codoped TiO_2_ the average size was estimated as 12 nm. The TEM images confirm that Ga–Zr-codoped TiO_2_ is nanoparticles and are approximately spherical in shape. The rings in SAED image fit with tetragonal and the TEM image shows the clear fringes with spacing of 0.24 nm corresponds with (110) plane of tetragonal TiO_2_. The scanning electron micrograph (SEM) of Ga–Zr-codoped TiO_2_ nanoparticles is displayed in Fig. 2 and the EDX of Ga–Zr-codoped TiO_2_ confirm the constituent elements (Fig. 2). The XRD peaks for Ag NPs at 2*θ* with interplanar reflections of 38.21° (1 1 1), 44.32° (2 0 0), 64.49° (2 2 0), 77.30° (3 1 1) and 81.44° (2 2 2) corresponds to face centered cubic crystal (FCC) (JCPDS no. 87-0597).^47–58^ The crystal size of Ag NPs is estimated as 17.32 nm and the surface area as 61.1 m^2^ g^−1^. The fringe distance 2.33 Å is calculated with spacing between (111) plane of FCC silver crystal and SAED confirm its crystalline nature. The clear diffraction circles along with bright spots in SAED pattern are identified to (111), (200), (220), (311) and (222) planes of FCC silver which is in correspondence with XRD results. The TEM images confirmed the spherical nature of Ag NPs (Fig. 3). The composition of Ga–Zr-codoped TiO_2_ and Ag NPs was examined by XPS which reveal Ga, Ti, Zr, O and C peaks (Fig. 4). The Gaussian peaks located at 181.3 (Zr 3d_5/2_) and 184.2 eV (Zr 3d_3/2_) and 458.8 (Ti 2p_3/2_) and 463.7 eV (Ti 2p_1/2_) confirmed Zr^4+^ (ref. 59) and Ti^4+^ (ref. 60) in Ga–Zr-codoped TiO_2_. The Ga 2p_3/2_ peak located at 1116.0 eV shows that gallium ions present as Ga^3+^ in Ga–Zr-codoped TiO_2_. Because of radii similarity, Ga^3+^ ions are incorporated into Ti^4+^ sites leading to a substitution doping.^61^ The signals at 530.1 (chemisorbed oxygen) and 531.8 (O^2−^ ions in TiO_2_ matrix) confirmed the presence of oxygen.^62,63^ The 366.8 (Ag 3d_5/2_) and 372.0 eV (Ag 3d_3/2_) signals corresponds with metallic silver:^64^ 525.0 and 530.9 eV signals reveal that O 1s profile is asymmetric in Ag NPs collected from *M. elengi* fruit pericarp (Fig. S2†). The composition of silver embedded Ga–Zr-codoped TiO_2_ film reveal that the red shifted peaks were observed for Ag, Ga, Ti, Zr, O and C on comparison with Ga–Zr-codoped TiO_2_ (Fig. 4). The Gaussian peaks located at 182.8 (Zr 3d_5/2_) and 185.9 eV (Zr 3d_3/2_) and 459.1 (Ti 2p_3/2_) and 467.0 eV (Ti 2p_1/2_) confirmed Zr^4+^ (ref. 59) and Ti^4+^ (ref. 60) in Ga–Zr-codoped TiO_2_. The Ga 2p_3/2_ peak located at 1119.0 eV shows Ga ions present as Ga^3+^ in Ga–Zr-codoped TiO_2_ (ref. 61) and the signals at 530.3 and 532.8 eV reveal that O 1s profile is asymmetric.^62,63^ The 368.9 (Ag 3d_5/2_) and 373.8 eV (Ag 3d_3/2_) signals corresponds with metallic silver.^64^ When the hole injection material was deposited its energy level was stabilized due to electric double layer formation and red shifted.^65,66^ The DRS of sol–gel synthesized Ga–Zr-codoped TiO_2_ presented in term of *F*(*R*) deduced from reflectance (*R*). The Kubelka–Munk algorithm [*F*(*R*) = (1 − *R*)^2^/2*R*] of Ga–Zr-codoped TiO_2_ is shown in Fig. 5. The absorption edge of TiO_2_ is 387 nm which corresponds to 3.20 eV band gap. Remarkable red shifts (420 nm) were obtained for doped Ga–Zr-codoped TiO_2_ thin film.^67^ UV-visible measurement of synthesized Ag NPs show surface plasmon absorption at 409 nm. Few percentage of co-doping effect induced shocking and causes significant red shift on comparison with single metal dopant effect.^68,69^ Higher absorption coefficient and well defined edge are desirable for an efficient optical absorber. The PL spectra of in Ga–Zr-codoped TiO_2_ show emission at 371 and 556 nm (Fig. 5).

### 3.2. Optical and electrochemical properties of emissive layers

The UV-vis absorption (*λ*_abs_) spectra of heteroleptic iridium(iii) complexes namely, Ir(fni)_3_, Ir(bbt)_2_(acac) and Ir(tfpdni)_2_(pic) in CH_2_Cl_2_ along with their corresponding free ligands fni, bbt and tfpdni, respectively (Fig. 5). The intensified absorption peaking of heteroleptic iridium complexes (Ir(tfpdni)_2_(pic) – 267 nm and Ir(bbt)_2_(acac) – 282 nm) is at the same energy level of free ligands bbt and tfpdni, arises from π–π* transition of cyclometalated ligands. For homoleptic complex Ir(fni)_3,_ the absorption at 278 nm is ascribed to spin-allowed ligand-centered transition of imidazole fragment. The other two bands (Ir(tfpdni)_2_(pic) – 376 & 392 nm and Ir(bbt)_2_(acac) – 330 & 411 nm) [Ir(fni)_3_ – 349 & 389 nm] are assigned to MLCT transitions [^1^MLCT ← S_0_; and ^3^MLCT ← S_0_]. The intensity of ^3^MLCT ← S_0_ transition is in closest with that of ^1^MLCT ← S_0_ transition which shows that ^3^MLCT ← S_0_ transition are strongly symmetry allowed by spin–orbit coupling.^70–77^

**Fig. 6:**
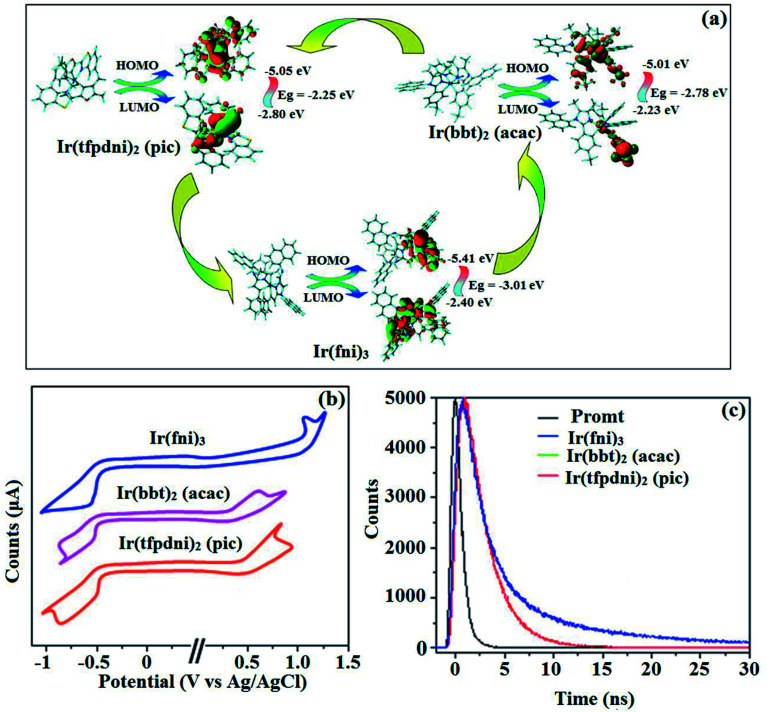
(a) Frontier molecular orbital contour map; (b) cyclic voltammogram and (c) lifetime spectra of Ir(fni)_3_, Ir(tfpdni)_2_(pic) and Ir(bbt)_2_(acac).

The three emissive complexes Ir(fni)_3_, Ir(bbt)_2_(acac) and Ir(tfpdni)_2_(pic) show strong luminescence both in solution and solid from their triplet manifold. The broad phosphorescence spectra of Ir(bbt)_2_(acac) and Ir(tfpdni)_2_(pic) show emission at 618 and 532 nm, respectively [Ir(fni)_3_ – 448 nm] (Fig. 5) and quantum yield (*Φ*) was measured as 0.68 and 0.81, respectively [Ir(fni)_3_ – 0.92]. The Franck–Condon-electronic transitions of emissive layer an displayed in Fig. 5 Ir(fni)_3_ and Ir(bbt)_2_(acac) and Ir(tfpdni)_2_(pic) in Fig. S3.† Generally, phosphorescence spectra from ligand-centered ^3^π–π* state is in vibronic whereas PL spectra from ^3^MLCT is in broad shape.^78–81^ Absence of vibronic emission spectra of iridium complexes [Ir(fni)_3_, Ir(bbt)_2_(acac) and Ir(tfpdni)_2_(pic)] supports the MLCT nature of emission which is confirmed by their phosphorescence life time of Ir(fni)_3_ (1.3 μs), Ir(bbt)_2_(acac) (1.8 μs) and Ir(tfpdni)_2_(pic) (2.7 μs) (Fig. 6). The broad emission spectra reveal that the excited triplet state of Ir(bpima)_2_(pic) and Ir(fni)_3_ possess dominant ^3^MLCT character.

**Fig. 7:**
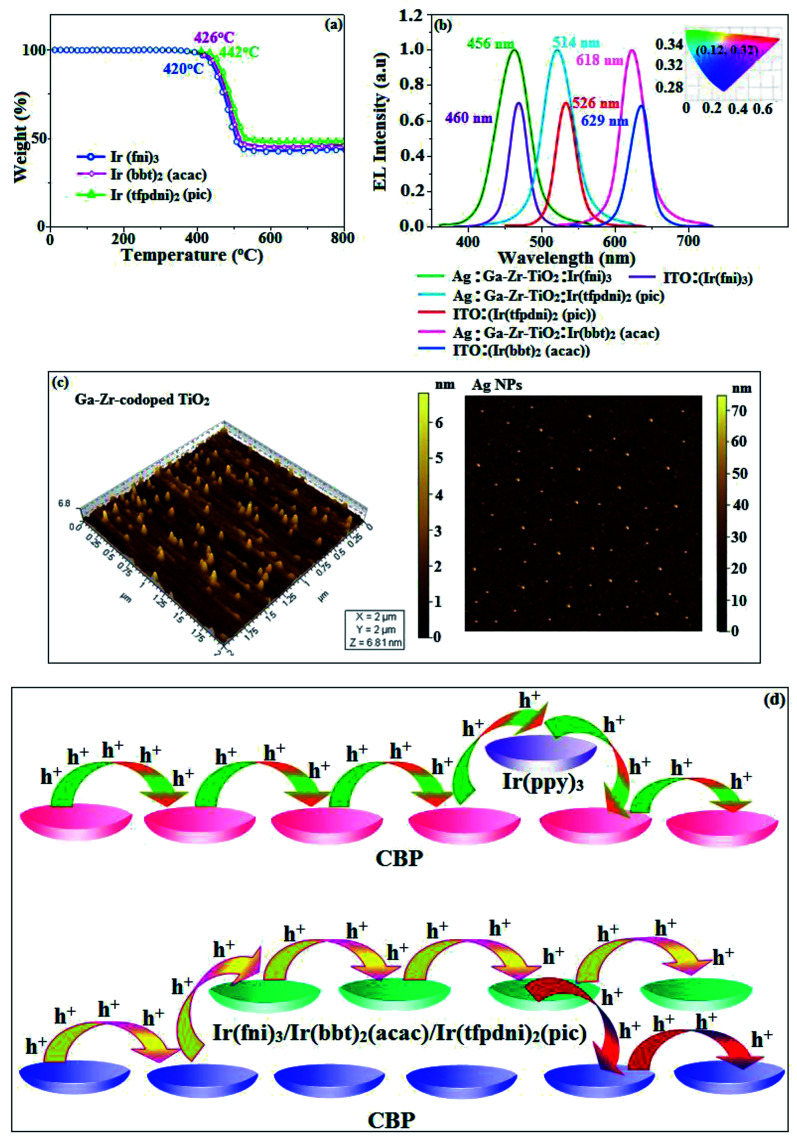
(a) TGA graph; (b) EL spectra (inset: CIE coordinates); (c) AFM images of Ir(fni)_3_, Ir(tfpdni)_2_(pic) and Ir(bbt)_2_(acac) and (d) schematic representation of (i) carrier trapping at Ir(ppy)_3_ and (ii) carrier hopping through CBP and Ir(fni)_3_/Ir(bbt)_2_(acac)/Ir(tfpdni)_2_(pic).

The electrochemical analysis of Ir(fni)_3_, Ir(bbt)_2_(acac) and Ir(tfpdni)_2_(pic) was employed with cyclic voltammetry with ferrocene/ferrocenium couple as internal reference and supporting electrolyte (0.1 M tetra(*n*-butyl)ammonium hexafluorophosphate: Fig. 6). The electrochemical stability of the complexes Ir(fni)_3_, Ir(bbt)_2_(acac) and Ir(tfpdni)_2_(pic) was confirmed by reversible one-electron oxidation wave. The HOMO energy of Ir(fni)_3_, Ir(bbt)_2_(acac) and Ir(tfpdni)_2_(pic) was measured as −5.41, −5.05 and −5.01 eV respectively,^82^ was obtained from oxidation potential and Fc/Fc^+^ redox couple energy [*E*_HOMO_ (eV) = −(*E*_ox_ + 4.8)] whereas the LUMO energy of Ir(fni)_3_, Ir(bbt)_2_(acac) and Ir(tfpdni)_2_(pic) was measured as −2.40, −2.80 and −2.23 eV which was deduced from *E*_LUMO_ = *E*_HOMO_ − 1239/*λ*_onset_ (Fig. 6 and Table 1). The HOMO orbital of Ir(tfpdni)_2_(pic) is localized on iridium, trifluoromethylbenzaldehydic and picolinate fragments whereas the LUMO orbital is majorly populated on trifluoromethylbenzaldehydic fragment. The HOMO of Ir(fni)_3_ is populated on iridium and fluorophenyl acrylaldehydic fragments and in LUMO the electron density is localized on naphthyl, iridium and fluorophenyl acrylaldehydic fragments. The HOMO orbital of Ir(bbt)_2_(acac) is majorly populated on iridium and 1,1,1,5,5,5-hexafluoropentane-2,4-dione fragments and partially on 2-aminobenzenethiol and benzofuran-2-carboxylic moieties whereas the LUMO orbital is partially populated on entire complex. The thermal characterization (*T*_d_) of Ir(fni)_3_, Ir(bbt)_2_(acac) and Ir(tfpdni)_2_(pic) were analyzed by TGA measurements to test its suitability for device fabrication. The TGA of Ir(fni)_3_, Ir(bbt)_2_(acac) and Ir(tfpdni)_2_(pic) exhibits high decomposition temperature (*T*_d5_) of 420, 442 and 426 °C, respectively (Fig. 7). The higher decomposition of blue, red and green emissive materials supports the suitability of these materials for fabrication of OLEDs and so it is expected that the synthesized iridium complexes Ir(fni)_3_, Ir(bbt)_2_(acac) and Ir(tfpdni)_2_(pic) will lower the turn on voltage in device performances. From the optimized geometry (Fig. 6) it was concluded that for Ir–C_av_ bond length is shorter than Ir–N_av_ bond length, Ir–N_av_ Ir(fni)_3_ (Ir–C_av_ – 2.02 Å < Ir–N_av_ – 2.10 Å), Ir(bbt)_2_(acac) (Ir–C_av_ – 2.01 Å < Ir–N_av_ – 2.07 Å) and Ir(tfpdni)_2_(pic) (Ir–C_av_ – 1.98 Å < Ir–N_av_ – 2.05 Å).^80,83^

**Table 1:** Optical and thermal properties of Ir(fni)_3_, Ir(bbt)_2_(acac) and Ir(tfpdni)_2_(pic)

Parameters	Ir(fni)_3_	Ir(tfpdni)_2_(pic)	Ir(bbt)_2_(acac)
Photophysical & thermal properties			
*λ*_ab_ (nm)	278 349 389	267 376 392	282 330 411
*λ*_em_ (nm)	448 452	506 532	603 618
*T*_d5_ (°C)	420	426	442
*Φ*	0.92	0.81	0.68
HOMO/LUMO (eV)	−5.41/−2.40	−5.01/−2.23	−5.05/−2.80
*E*_g_ (eV)	−3.01	−2.78	−2.25
*τ* (ns)	1.3	2.7	1.8
*k*_r_ × 10^8^ (s^−1^)	7.0	3.0	3.7
*k*_nr_ × 10^8^ (s^−1^)	0.9	0.7	1.8

### 3.3. Blue, green and red phosphorescent OLED performances

**Fig. 8:**
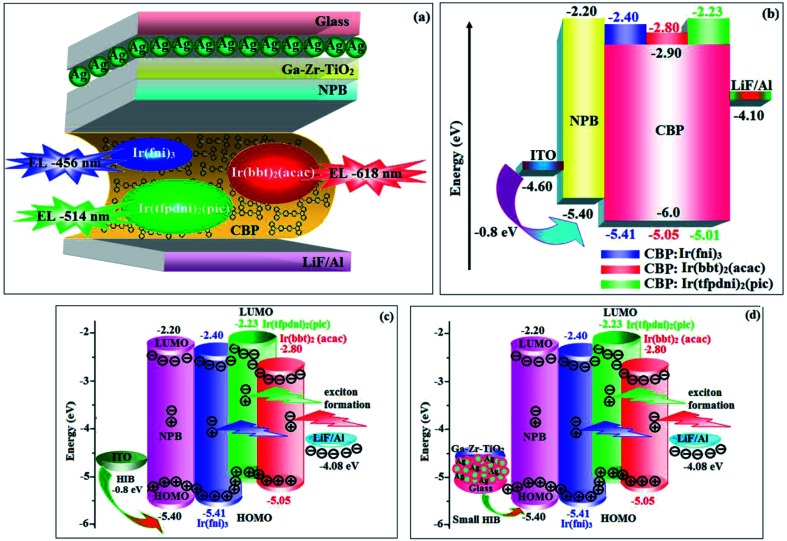
Device structure (a) glass/Ag/Ga–Zr-TiO_2_/NPB/CBP:Ir(fni)_3_ (or) CBP:Ir(bbt)_2_(acac) (or) CBP:Ir(tfpdni)_2_(pic)/LiF/Al; (b) ITO/NPB/CBP:Ir(fpi)_3_ (or) CBP:Ir(bbt)_2_(acac) (or) CBP:Ir(tfpdni)_2_(pic)/LiF/Al and their energy level diagrams (c) and (d).

**Fig. 9:**
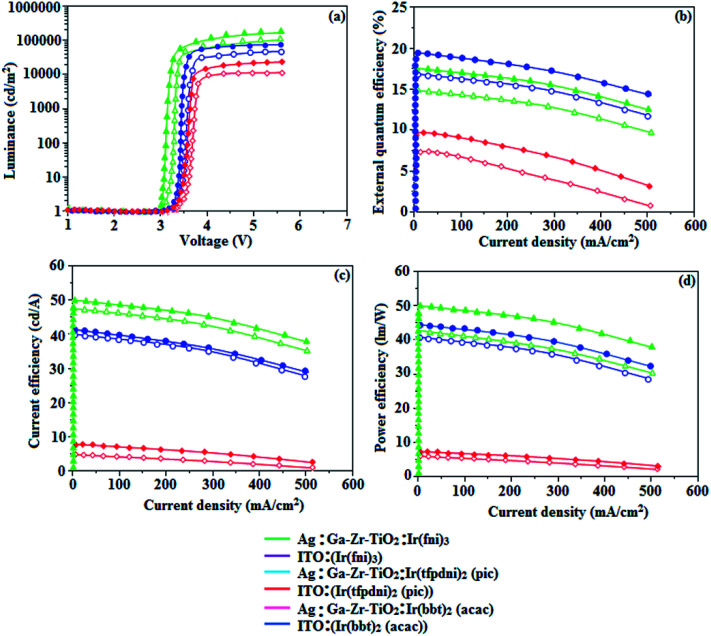
Electroluminescence performances: (a) luminance *versus* voltage; (b) external quantum efficiency *versus* current density; (c) current efficiency *versus* current density and (d) power efficiency *versus* current density of Ir(fni)_3_, Ir(tfpdni)_2_(pic) and Ir(bbt)_2_(acac).

The device fabrication was made using iridium(iii) complexes namely Ir(fni)_3_, Ir(tfpdni)_2_(pic) and Ir(bbt)_2_(acac) as blue, green and red emitters, respectively. The fabricated device structures and their energy level diagram are shown in Fig. 8. Furthermore, the *N*,*N*′-dicarbazolyl-4,4′-biphenyl (CBP) was employed as host material due to its wide energy gap and bipolar transport capability allowing balanced h^+^–e^−^ recombination in the emissive layer.^83,84^ High triplet energy (2.3 eV) of HTM [*N*,*N*′-bis(naphthyl)-*N*,*N*′-diphenyl-1,1′-biphenyl-4,4′-diamine (NPB)] facilitates high energy exciton confinement.^85–89^ The RMS (2.11 nm) of silver NPS embedded Ga–Zr-codoped TiO_2_ is higher than ITO (Fig. 7). The Ag NPs heat conductivity is higher than ITO which induced large grains on Ga–Zr-codoped TiO_2_ surface. The work function (*E*_F_) of Ga–Zr-codoped TiO_2_ surface with Ag NPs interlayer is expected to be stabilized than *E*_F_ of ITO. Hence, the interlayer of Ag NPs increase the conductivity and *E*_F_ on Ga–Zr-codoped TiO_2_ surface results enhancing the luminance of devices (Table 2). The current density and luminance of the devices increased with the incorporation of silver nanoparticles at the interface of glass:Ga–Zr-codoped TiO_2_ than reference devices II, IV and VI (Fig. 9). Among the blue devices I (456 nm: Fig. 7) and II (460 nm), device I exhibit maximum luminance (*L*) of 40 512 cd m^−2^ (ITO – 37 623 cd m^−2^), current efficiency (*η*_c_) of 41.3 cd A^−1^ (ITO – 40.5 cd A^−1^) and power efficiency (*η*_p_) of 43.1 lm w^−1^ (ITO – 39.8 lm w^−1^) with external quantum efficiency (*η*_ex_) of 19.4% (ITO – 16.9%) at driving voltage 3.2 V. Similar observations were found for green and red devices. Newly fabricated green device with emissive layer Ir(tfpdni)_2_(pic) show intensified emission at 514 nm (Fig. 7) and luminance of 46 435 cd m^−2^ (ITO – 40 986 cd m^−2^), current efficiency (*η*_c_) of 49.7 cd A^−1^ (ITO – 47.3 cd A^−1^), power efficiency (*η*_p_) of 48.6 lm w^−1^ (ITO – 41.4 lm w^−1^) and external quantum efficiency (*η*_ex_) of 17.5% (ITO – 14.9%). The red device (618 nm: Fig. 7) with emissive layer Ir(bbt)_2_(acac) show luminance of 8936 cd m^−2^ (ITO – 8043 cd m^−2^), current efficiency (*η*_c_) of 6.9 cd A^−1^ (ITO – 4.6 cd A^−1^), power efficiency (*η*_p_) of 5.7 lm w^−1^ (ITO – 4.9 lm w^−1^) and external quantum efficiency (*η*_ex_) of 9.3% (ITO – 6.9%) (Fig. 9). The devices I, III and V show intensified emission than reference devices (II, IV & VI) since the hole injection barrier is reduced by stabilizing the *E*_F_ by the incorporation of Ag NPs at the glass:Ga–Zr-codoped TiO_2_ interface results voltage reduction with intensified emission than the reference devices II, IV and VI.^39^ Since the embedded silver nanoparticles at glass:Ga–Zr-codoped TiO_2_ interface facilitate the holes to diffuse across the junction which results enhanced efficiencies: leakage of holes through emissive layer was decreased results balanced h^+^–e^−^ recombination in the emissive layer and thus the nonproductive hole current was removed. The larger interface area between the emitter and HTL layer enhanced the charge injection make effective electron–hole recombination results enhanced device performances.^89^ Moreover the surface-plasmon enhanced emission can strongly promote the external emission of devices I, III and V. At Ga–Zr-codoped TiO_2_ film, Ag NPs effectively excite such emission and enhanced the device efficiencies (I, II and V). Efficient electroluminescent performances were achieved from the fabricated devices (I, III and V) with silver nanoparticles incorporation at glass:Ga–Zr-codoped TiO_2_ interface. In CBP:Ir(ppy)_3_ (25 nm) based devices the carrier current decreases sharply since the carrier may undergo deep trapping at Ir(ppy)_3_ HOMO orbital. However, in CBP:Ir(fni)_3_ (25 nm); CBP:Ir(bbt)_2_(acac) (25 nm); CBP:Ir(tfpdni)_2_(pic) (25 nm) based devices, the carrier current increased which may be attributed to the effect of direct injection into the dopant HOMO levels and the hopping transport thorough Ir(fni)_3_/Ir(bbt)_2_(acac)/Ir(tfpdni)_2_(pic) and dopant sites (Fig. 7). Overall, the efficiencies of the tested blue, green and red PHOLEDs indicate that the electroluminescent efficiencies of devices based on different anodes are comparable and the electroluminescent performances of OLEDs with various anodes are displayed in Table 3.^85–90^ The performances of the Ga–Zr-codoped TiO_2_-based OLEDs were not inferior to those of other previously reported efficiencies. This outstanding performance manifests the great potential of Ga–Zr-codoped TiO_2_ film as an alternative anode for OLEDs.

**Table 2:** Comparative device efficiencies of Ag/Ga–Zr-TiO_2_ and ITO green, red and blue devices

Device parameters	Ir(fni)_3_		Ir(tfpdni)_2_(pic)		Ir(bbt)_2_(acac)	
	Ag/Ga–Zr-TiO_2_ (I)	ITO (II)	Ag/Ga–Zr-TiO_2_ (III)	ITO (IV)	Ag/Ga–Zr-TiO_2_ (V)	ITO (VI)
*V*_on_ (V)	3.2	3.2	3.0	3.0	3.3	3.3
*L* (cd m^−2^)	40 512	37 623	46 435	40 986	8936	8043
*η*_ex_ (%)	19.4	16.9	17.5	14.9	9.3	6.9
*η*_c_ (cd A^−1^)	41.3	40.5	49.7	47.3	6.9	4.6
*η*_p_ (lm W^−1^)	43.1	39.8	48.6	41.4	5.7	4.9
CIE (*x*, *y*)	(0.15, 0.10)	(0.16, 0.10)	(0.12, 0.32)	(0.12, 0.33)	(0.68, 0.30)	(0.68, 0.32)
EL (nm)	456	460	514	526	618	629

**Table 3:** External quantum efficiency of OLEDs with various anodes

Anode	Emissive materials/color	EQE (%)	Reference
Zr:ZnO	Alq_3_/green	0.87	85
In:ZnO	*Fac*-Ir(ppy)_3_/green	13.2	86
Ga:ZnO	FIrpic/blue	1.52	87
NPs	FIrpic/blue	8.2	88
Ga–Ti-ZnO	Ir(piq)_2_acac/red	9.1	88
	*Fac*-Ir(ppy)_3_/green	14.5	89
	FIrpic/blue	19.0	89
Ag/Ga–Ti-ZnO	Ir(bpima)_2_(pic)/blue	19.2	90
	Ir(fpi)_3_/green	15.6	90
Ag/Ga–Zr-TiO_2_	Ir(fni)_3_/blue	19.4	This work
	Ir(tfpdni)_2_(pic)/green	17.5	This work
	Ir(bbt)_2_(acac)/red	9.3	This work

## 4. Conclusion

The coupling of surface plasmonic and hole injection ability exerted by Ag NPs enhanced the fabricated device efficiency. Enhanced efficiencies have been obtained by incorporating Ag NPs at glass:Ga–Ti-codoped TiO_2_ interface to avoid altering the hole mobility on Ga–Ti-codoped TiO_2_ surface. Among the blue devices I (456 nm) and II (460 nm), device I exhibit maximum luminance (*L*) of 40 512 cd m^−2^ (ITO – 37 623 cd m^−2^), current efficiency (*η*_c_) of 41.3 cd A^−1^ (ITO – 40.5 cd A^−1^) and power efficiency (*η*_p_) of 43.1 lm w^−1^ (ITO – 39.8 lm w^−1^) with external quantum efficiency (*η*_ex_) of 19.4% (ITO – 16.9%) at driving voltage 3.2 V. Similar observations were found for green and red devices. Newly fabricated green device with emissive layer Ir(tfpdni)_2_(pic) show intensified emission at 514 nm and luminance of 46 435 cd m^−2^ (ITO – 40 986 cd m^−2^), current efficiency (*η*_c_) of 49.7 cd A^−1^ (ITO – 47.3 cd A^−1^), power efficiency (*η*_p_) of 48.6 lm w^−1^ (ITO – 41.4 lm w^−1^) and external quantum efficiency (*η*_ex_) of 17.5% (ITO – 14.9%). The red device (618 nm) with emissive layer Ir(bbt)_2_(acac) show luminance of 8936 cd m^−2^ (ITO – 8043 cd m^−2^), current efficiency (*η*_c_) of 6.9 cd A^−1^ (ITO – 4.6 cd A^−1^), power efficiency (*η*_p_) of 5.7 lm w^−1^ (ITO – 4.9 lm w^−1^) and external quantum efficiency (*η*_ex_) of 9.3% (ITO – 6.9%). The outcome of present investigation reveals the potential advantages by the use of Ga–Zr-codoped TiO_2_ as an alternative OLED anode in terms of higher efficiency, low voltage and luminance. The superior characteristics of this tailored silver embedded Ga–Zr-codoped TiO_2_ anode points toward the replacement of ITO anodes in future OLED applications.

## Conflicts of interest

There are no conflicts to declare.

## Supplementary Material

RA-009-C9RA01025D-s001
