# Supplementary material for: Promotional effect of silver nanoparticle embedded Ga–Zr-codoped TiO2 as an alternative anode for efficient blue, green and red PHOLEDs
Source: RSC Adv. 2019 May 3;9(24):13664–76. doi: 10.1039/c9ra01025d (PMC9063942; doi:10.1039/c9ra01025d)

Electronic Supplementary Material (ESI) for RSC Advances.
This journal is © The Royal Society of Chemistry 2019

Supporting Information

Promotional effect of silver nanoparticles embedded Ga-Zr-codoped TiO_2 as an alternative anode for an efficient blue, green and red PHOLEDs

Jayaraman Jayabharathi*, Pavadai Nethaji, Venugopal Thanikachalam

Department of Chemistry, Annamalai University, Annamalainagar 608 002, Tamilnadu, India

Figure S1: HPLC of [Ir(fpi)$_3$], [Ir(tfpdni)$_2$ (pic)] and [Ir(bbt)$_2$(acac)].

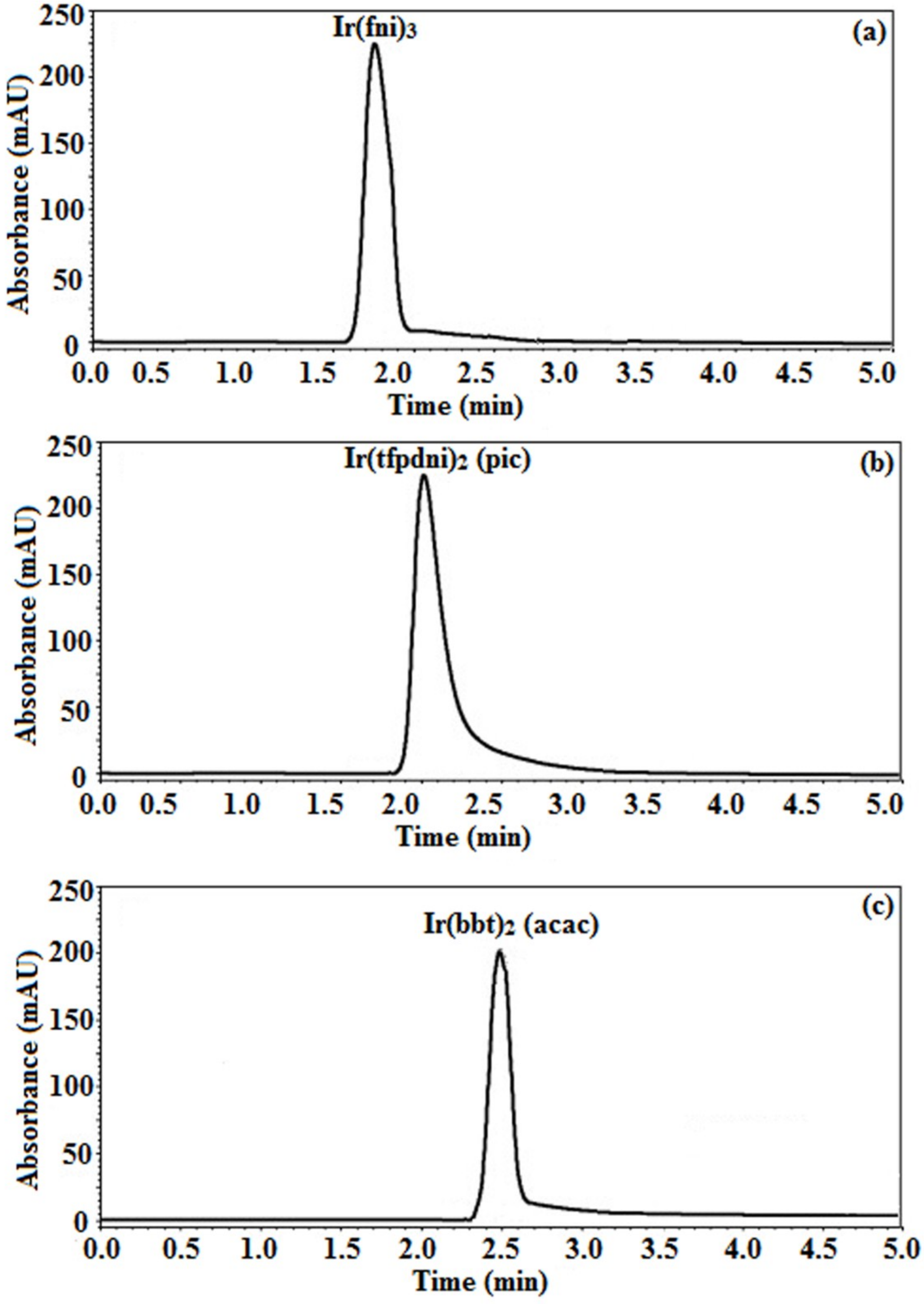

Figure S2. X-ray photoelectron spectra (XPS) of Ga-Zr-codoped TiO_2 and Ag/Ga-Zr-codoped TiO_2 film.

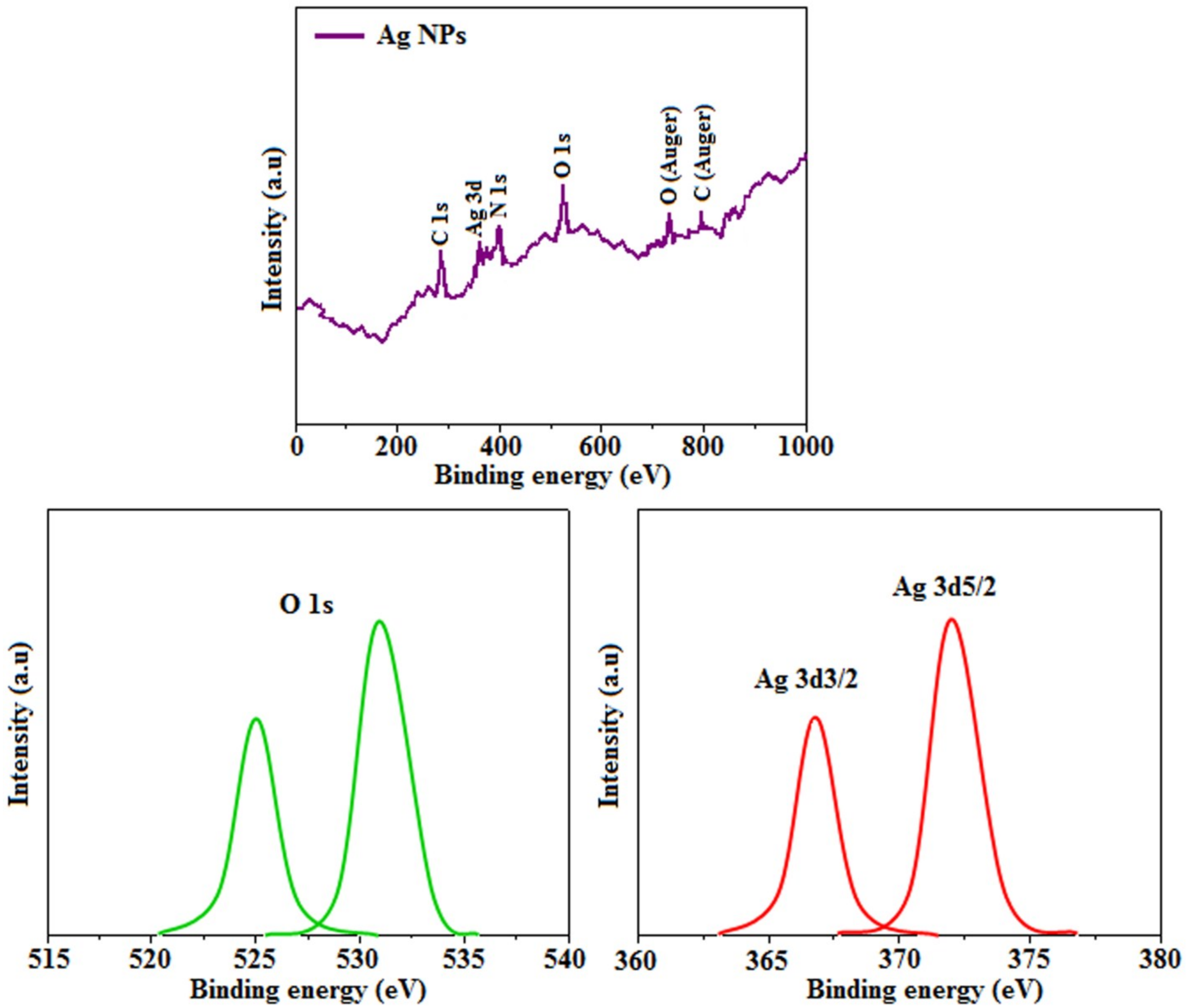

Figure S3. Franck - Condon electronic transitions of Ir(tfpdni)$_2$ (pic) (a) and Ir(bbt)$_2$ (acac) (b).

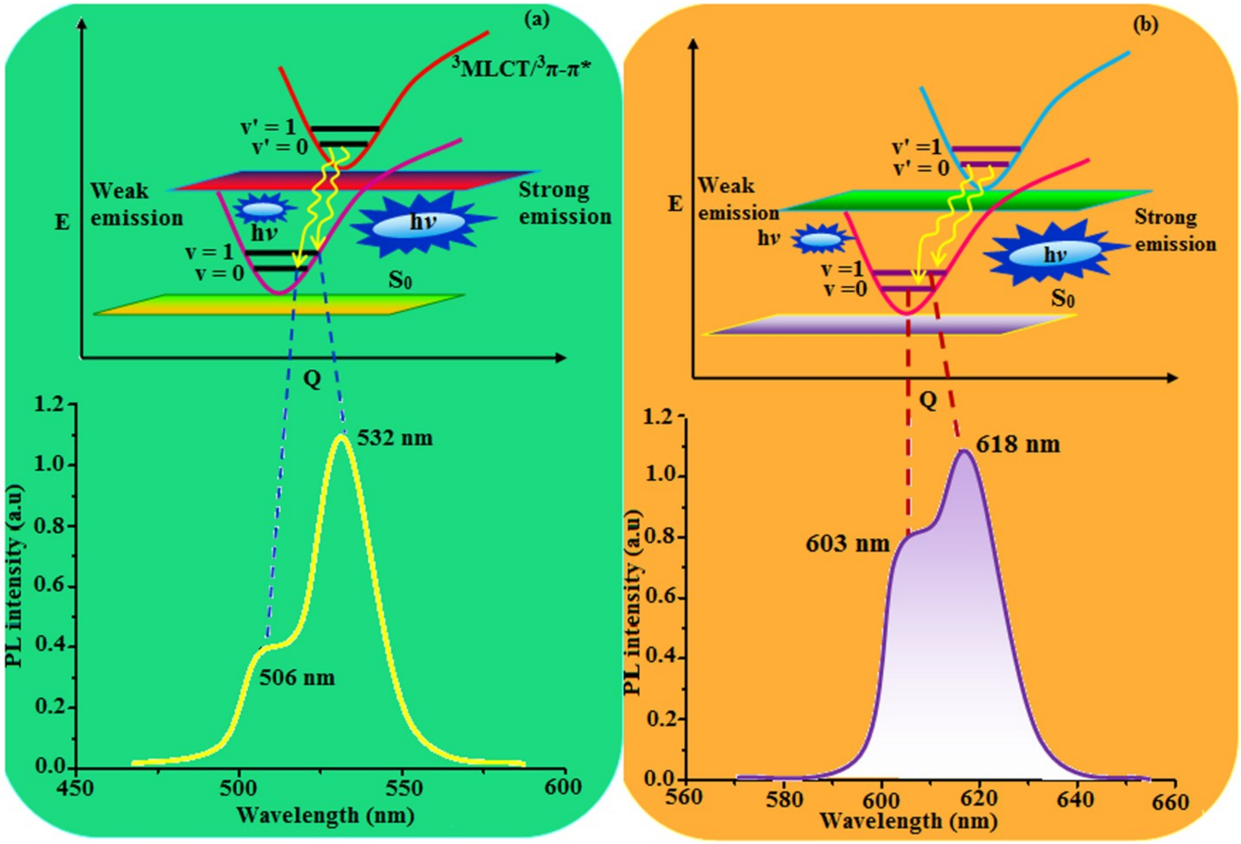

Supplement: RA-009-C9RA01025D-s001 [file RA-009-C9RA01025D-s001.pdf]
